# Effects of core stabilization exercise and strengthening exercise on proprioception, balance, muscle thickness and pain related outcomes in patients with subacute nonspecific low back pain: a randomized controlled trial

**DOI:** 10.1186/s12891-021-04858-6

**Published:** 2021-11-30

**Authors:** Su Su Hlaing, Rungthip Puntumetakul, Ei Ei Khine, Rose Boucaut

**Affiliations:** 1 Human Movement Sciences, School of Physical Therapy, Faculty of Associated Medical Sciences, Khon Kaen University, 123 Mittraphap Rd, Muang District, Khon Kaen, 40002 Thailand; 2 Research Center in Back, Neck, Other Joint Pain and Human Performance, Khon Kaen University, 123 Mittraphap Rd, Muang District, Khon Kaen, 40002 Thailand; 3 School of Physical Therapy, Faculty of Associated Medical Sciences, Khon Kaen University, 123 Mittraphap Rd, Muang District, Khon Kaen, 40002 Thailand; 4 Department of Radiology, Yangon Orthopedic Hospital, Kyee Myin Daing Township, Yangon, 11101 Myanmar; 5 University of South Australia: Allied Health and Human Performance, Adelaide, SA 5001 Australia

**Keywords:** Subacute low back pain, Stabilization exercise, Joint repositioning error, Ultrasound

## Abstract

**Background:**

Therapeutic exercises are used in clinical practice for patients with low back pain (LBP). Core stabilization exercises can retrain the important function of local trunk muscles and increase the accuracy of the sensory integration process for stability of the spine in individuals with LBP. The aim of this study was to compare the effects of two different exercise regimes, Core stabilization exercises (CSE) and Strengthening exercise (STE), on proprioception, balance, muscle thickness and pain-related outcomes in patients with subacute non-specific low back pain (NSLBP).

**Methods:**

Thirty-six subacute NSLBP patients, [mean age, 34.78 ± 9.07 years; BMI, 24.03 ± 3.20 Kg/m^2^; and duration of current pain, 8.22 ± 1.61 weeks], were included in this study. They were randomly allocated into either CSE (*n* = 18) or STE groups (n = 18). Exercise training was given for 30 min, three times per week, for up to 4 weeks. Proprioception, standing balance, muscle thickness of transversus abdominis (TrA) and lumbar multifidus (LM), and pain-related outcomes, comprising pain, functional disability and fear of movement, were assessed at baseline and after 4 weeks of intervention.

**Results:**

The CSE group demonstrated significantly more improvement than the STE group after 4 weeks of intervention. Improvements were in: proprioception [mean difference (95% CI): − 0.295 (− 0.37 to − 0.2), effect size: 1.38, (*p* <  0.001)], balance: single leg standing with eyes open and eyes closed on both stable and unstable surfaces (*p* <  0.05), and percentage change of muscle thickness of TrA and LM (*p* <  0.01). Although both exercise groups gained relief from pain, the CSE group demonstrated greater reduction of functional disability [effect size: 0.61, (*p* <  0.05)] and fear of movement [effect size: 0.80, (*p* < 0.01)]. There were no significant adverse effects in either type of exercise program.

**Conclusion:**

Despite both core stabilization and strengthening exercises reducing pain, core stabilization exercise is superior to strengthening exercise. It is effective in improving proprioception, balance, and percentage change of muscle thickness of TrA and LM, and reducing functional disability and fear of movement in patients with subacute NSLBP.

**Trial registration:**

Thai Clinical Trial Registry (TCTR20180822001; August 21, 2018).

**Supplementary Information:**

The online version contains supplementary material available at 10.1186/s12891-021-04858-6.

## Background

Globally, low back pain (LBP) is a major contributor to disability; it is both a common health and socioeconomic challenge. A Global Burden of Disease 2010 study revealed that LBP ranked as the most severe disability in terms of years lived with disability [1]. Additionally, Hoy and colleagues (2012) estimated that the mean point prevalence, 1-year prevalence, and lifetime prevalence of LBP were (18.3%), (38.0%), and (38.9%), respectively [2]. Approximately 30% of the total number of patients attending physiotherapy departments suffer from LBP, and it is the most prevalent musculoskeletal disorder in Myanmar [data from the physiotherapy outpatients department at Yangon Orthopedic Hospital, 2017–2018].

Low back pain causes pain localized between the costal margins and the inferior gluteal folds, and may exist with or without lower extremity pain [3]. More than 85% of LBP cases are categorized as nonspecific LBP (NSLBP) with no identifiable cause or pathology [4, 5]. Postural control, which is essential for executing functional activities, is diminished in patients with NSLBP [6]. Postural control is a complex neuromuscular process that depends on sensory input from the visual, vestibular, and somatosensory systems [6–8].

Proprioception is the main component of the somatosensory system and provides integration of sensory input, central processing and motor output for postural control [9]. A systematic review revealed that proprioception is more impaired in patients with chronic LBP than in healthy controls [10]. Proprioceptive deficits causing alteration in lumbar spine motion may be a potential mechanism for LBP [11, 12]. Reduced proprioceptive acuity may decrease the skill of achieving and retaining a neutral spinal posture, coordination of muscles, and thereby lessen balance control in populations with LBP. Reduced postural control in LBP patients could lead to pain, disability and recurrences of injury. So decreased proprioception in people with LBP may lead to impairment in sensorimotor control and that has been proposed to be either or both a result and a cause of their pain [13, 14].

Poor muscle coordination (including decreased intrinsic postural muscle activity, increased superficial muscle activity, and lack of spinal flexibility), and poor muscle recruitment patterns [15, 16] may alter the normal effective stability of the spine in patients with LBP [17, 18]. Most LBP patients have impaired fine-tuning of neuromuscular control and rigid spinal posture [19]. Hlaing and coworkers (2020), recently reported that balance control and proprioception are reduced in patients with subacute NSLBP when compared with healthy control subjects [14]. They also found that reduced proprioception is correlated with impaired balance. Possible mechanisms for this may be changes in function and structure throughout the nervous system that affect sensorimotor control [14].

Lumbar multifidus (LM), the main trunk stabilization muscle, may have reduced efficiency 24 h after the onset of acute LBP [20, 21]. Changes in muscle morphology may be localized to the injured part in the subacute stages and more generalized in the chronic stages of LBP [22]. In patients with chronic LBP, motor adaptation to compensate for pain may alter the distribution of activity within or between synergistic muscles, change sensory function, and alter the excitability and organization of the motor cortex and motor response planning. These changes may increase the load on tissues, cause tissue irritation and structural change over time and lead to further dysfunction [23]. Thus, early initiation of exercise is important to foster optimal recovery. A prior review proposed that effective interventions for patients with subacute LBP are important for the prevention of transition to chronic pain conditions [24].

Several forms of therapeutic exercises are used in clinical practice for patients with LBP. Core stabilization exercises (CSE), based on the motor learning approach, emphasize the co-activation of the transversus abdominis (TrA) and LM muscles. These deep stabilization muscles attach to the thoracolumbar fascia, create a stiffening effect in the lumbar spine by increasing intra-abdominal pressure, and provide segmental stability to the spine [25]. In addition, CSE can reverse pain-related restructuring in the motor cortex, enhance muscle behavior and retrain the important function of local trunk muscles for neuromuscular control of spinal stability [26]. Stabilization exercises may reduce pain and disability, improve proprioception, successfully modify postural impairments, and improve the stability index in patients with LBP [27–30].

Strengthening exercises (STE) are commonly used to treat patients with LBP. Strengthening exercises activate superficial trunk muscles that provide shock absorption of loads and are appropriate for patients with subacute or chronic NSLBP [31, 32]. These exercises aim to increase strength and control of the global trunk muscles to improve general spinal stability. These exercises could decrease pain and physical disability and increase trunk muscle activity in patients with NSLBP [32]. In the ankle, Docherty and colleagues (1998) suggest that strength training can increase both strength and joint position sense [33]. Strengthening exercise programs may increase gamma motor activity, improve the central mechanisms of motor control, or produce a combination of central and spindle mechanisms [34, 35]. No previous study has reported the effects of strengthening exercises on proprioception related to either the subacute or chronic stages of LBP.

As far as we know, only one study to date has evaluated the effect of CSE on proprioception in patients with subacute NSLBP compared with a control group. The findings demonstrated that a CSE program can reduce pain and improve proprioception compared with a control group [36]. It may be postulated that low-threshold recruitment of TrA and LM muscles can increase proprioception by providing efficient motor integration [37–39].

Prior research comparing the effects of CSE and STE on trunk muscle activation and stability index has mostly been undertaken in patients with chronic LBP, producing conflicting results [40, 41]. In patients with subacute LBP, the comparison of the effects of CSE and STE in terms of changes in proprioception, balance and muscle thickness of TrA and LM have not been scientifically confirmed to date.

Therefore, our primary purpose was to compare the effects of CSE and STE on proprioception in patients with subacute NSLBP. Our secondary purpose was to compare the effects of CSE and STE on balance, muscle thickness of TrA and LM and pain-related outcomes in patients with subacute NSLBP.

## Methods

An assessor-blinded randomized controlled trial was approved by the Ethics Committee of Khon Kaen University, Thailand (HE 612259) and the University of Public Health, Myanmar. The study proposal followed the CONSORT checklist and was registered at clinicaltrials.in.th (registration number: TCTR20180822001; August 21, 2018). It was conducted at the Physiotherapy Department, Yangon Orthopedic Hospital, Myanmar between December 2018 and April 2019. The intentions and processes of the study were explained to the eligible participants, and they were asked to sign an informed consent form before their participation.

### Participants

The eligibility criteria of participants for the current study included: age 20–50 years old, subacute NSLBP (6–12 weeks), no radiating leg pain, with moderate pain (VAS: 3–7) and a disability score of 19% or greater as evident from the modified Oswestry Disability Questionnaire (MODQ).

The postural control system can also be affected by aging, (decline in muscle strength, sensory functioning, or in speed of sensorimotor responses) and deteriorates from the age of 50 onwards [42, 43]. Balance may decline beyond 50 years of age due to the aging process, so, the upper age of participants in this study was limited to 50 years old. In the present study, patients with a pain score over 3/10 and with no more than 7/10 pain were selected because the outcome measures used in our study may not be suitable for patients with severe pain, especially the task of single leg standing [44].

Participants were excluded if they had: neuromuscular disorders, spine or other joint deformity, recent lower extremity injuries (within 6 months), brain injury, pregnancy, body mass index > 30, conditions that affect balance (drugs, alcohol consumption, visual and vestibular disorders), or compromised exercise performance (hypertension, ischemic heart disease, diabetes mellitus).

Sample size was calculated in relation to the primary outcome of the study: “joint repositioning error”, with the mean difference of (μ_1_ – μ_2_ = 0.35 points) and a pooled variance estimation (σ^2^ = 0.15) between the CSE and STE groups from the pilot study with 80% power and significance level set at α = 0.05. An initial sample size of 13 in each group was required by calculation using the formula: n = (z_α/2_ + z_β_)^2^ × σ^2^/(μ_1_ – μ_2_)^2^, where, we set z_α/2_ = 1.96 and z_β_ = 1.28. Allowing for a dropout rate of 15%, at least 36 participants (18 in each group) were recruited in this study. All eligible participants were randomly assigned to either the CSE or STE group (allocation ratio 1:1).

Baseline assessment of the outcome variables was performed by one physical therapist with 20 years of work experience and one Senior Consultant Radiologist after gaining patient consent. Another physical therapist with 7 years of working experience assigned the participants to either the CSE group or the STE group using a random allocation with a block size of six. Pre-generated random assignment schemes were enclosed in sealed envelopes and each participant chose their preferred envelope. Participants were asked not to participate in other treatment during the study period.

### Intervention procedure

Participants completed the exercise program for 30-min sessions, with three sessions per week for up to 4 weeks. In this study, 8–10-week CSE or STE programs were shortened to 4 weeks as our pilot study demonstrated that proprioception improvement occurs after 4 weeks of the exercise program. Each exercise was repeated 10 times, with 10 s holds, followed by a five-minute rest interval. The researcher (SSH) trained participants in the CSE group with the CSE program and the other experienced physical therapist trained participants in the STE group with the STE program. Participants in the CSE and STE groups completed either the CSE or STE training program. Participants were asked to perform their exercise routine as daily home exercises for 15 min. Other exercises were not permitted. Participants were asked to record details of their exercise practice on a log sheet, over the study period, to monitor their compliance. Additionally, the researcher made a phone call to all participants every week to encourage them to continue their home exercises during the study period.

### Core stabilization exercise

Participants in the CSE group received CSE training using the treatment approach described by Puntumetakul et al. (2013) with the exercise period shortened from10 weeks to 4 weeks [45]. Details of the CSE program are described in Additional file 1: Appendix 1.

There were two stages of CSE. In the first stage (weeks 1–2), the treatment emphasis was on isolating low-load activation of the TrA and LM muscles with an abdominal drawing-in maneuver (ADIM) technique. Subsequent training involved co-contraction of these muscles in low load conditions. To activate TrA in the first week, the participants were positioned in prone lying on a bed with a small pillow placed under their ankles. A pressure biofeedback device (Chattanooga Australia Pty Ltd., Brisbane, QLD, Australia) was used to provide visual feedback and was set at 70 mmHg, it was placed under the participants’ lower abdomen. The participants were asked to draw their lower anterior abdominal wall “up-and-in” towards the spine. If they performed the exercise successfully, pressure was lowered 6 to 10 mmHg. Isolated activation of LM was stimulated by raising the contralateral arm when performing ADIM seated on a chair. Manual contact provided feedback for LM activation. In the second week, co-contraction of the TrA and LM muscles with controlled movements of lower extremities was performed in supine lying and sitting positions.

The second stage (weeks 3–4), emphasized increasing the accuracy and duration of exercise. Co-contraction of the TrA and LM muscles with controlled movements of upper and lower extremities was performed and progressed to high load positions. Co-contraction of the TrA and LM muscles was performed while sitting on a balance board and while lying supine in the third week. In the fourth week of the exercise program, co-contraction of the TrA and LM muscles was performed in the quadruped position and standing.

During exercise performance, participants were trained to self-monitor by palpating for the contraction of TrA and LM. For contraction of TrA, their index and middle fingers palpated the area 2 cm medial to their anterior superior iliac spines, and for LM, participants placed their index and middle fingers near their L_5_ spinous process. The accuracy of contractions of the TrA and LM muscles was re-evaluated at every exercise session by the researcher physiotherapist.

### Strengthening exercise

Participants in the STE group underwent the STE program, which activated the back and abdominal muscles for extension and flexion, respectively. This program was adapted from that described by Koumantakis et al. (2005), with the exercise duration shortened from 8 to 4 weeks [32]. The program progressed based on individual performance, from lying to the quadruped position. The exercise program for the strengthening exercises is described in Additional file 1: Appendix 2.

In the first week, the participants were asked to train their upper abdominal muscles by performing partial sit-ups from the supine position with knees bent (crook lying) and to train their back extensors in the prone lying position by raising their trunk. In the second week, the participants were asked to undertake lower limb movement while training their upper abdominals and performing bridging from the supine lying position to train their back extensors.

The participants were asked to perform hip lifts in the side-lying position to train their oblique abdominal muscles and perform extension of one leg in the quadruped position to train back extensor muscles during the third week of the exercise program. In the 4 week, participants were asked to perform full abdominal crunches from the supine position and alternate arm and leg lifting from the quadruped position to train the back extensor muscles.

### Outcome measures

Proprioception was the primary measure in this study, and balance, muscle thickness of TrA and LM, and pain-related outcomes were used as secondary measures. All outcome measures were evaluated at baseline and 4 weeks after the intervention by two assessors who did not know the participant group’s assignment and intervention. An experienced physical therapist assessed proprioception, balance, and pain-related outcomes. Researcher (EEK) performed Rehabilitative Ultrasound Imaging (RUSI) using standardized tools to assess TrA and LM muscle thickness.

### Primary outcome

#### Proprioception

**Fig. 1:**
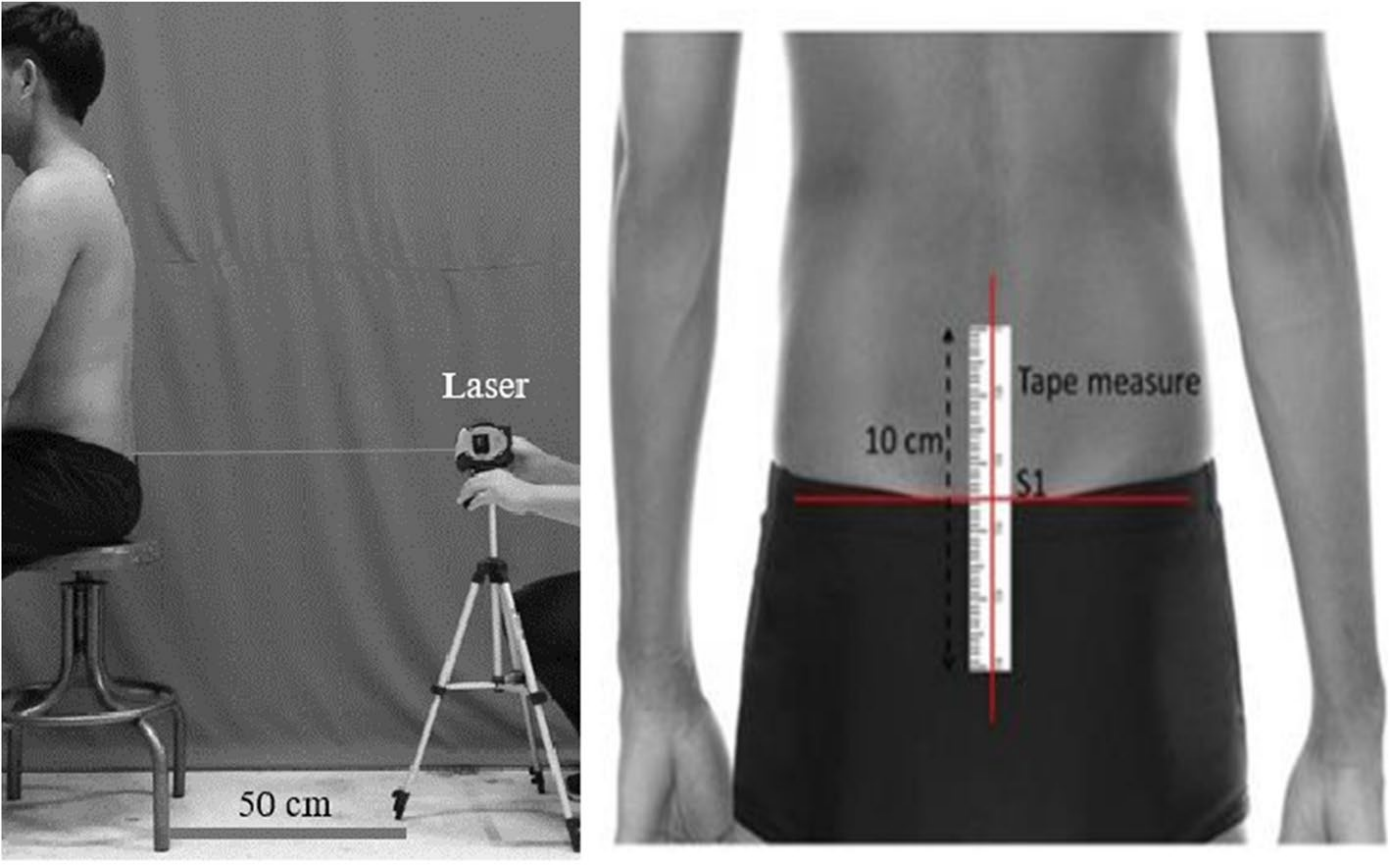
Measurement position for joint repositioning error. A laser pointer was put 50 cm behind the participants and pointed on the center of the 10 cm tape measure that positioned on first sacral segment (S_1_)

Proprioception was assessed with joint repositioning error as a primary outcome (Fig. 1). The method used followed the process described by Puntumetakul et al., 2018 [36]. “The participant was in the sitting position (90° hips and knees) with feet on the ground, hands on thighs and the examiner guided the participant into the neutral lumbar spine position. As the start point of measurement, the center of the 10-centimeter tape-measure was positioned on sacral segment 1 (S_1_). A laser pointer with a stable base was sited directly on the start point. The examiner instructed the participant to remember the target position, and then to perform the maximum anterior and posterior pelvic tilt twice, maintaining five seconds in each position,” and then returning to the neutral target position [36]. Deviance from the start point was measured in centimeters as a joint repositioning error. Feedback about any error was not given to the participant. Prior to the assessment, the participants practiced the repositioning test twice. This examination procedure was executed three times with 1 minute rest intervals. The mean values were used for analysis.

Repositioning error can be described by the absolute error, the constant error, and the variable error. We chose to calculate absolute error as it reflects accuracy [46], represents error magnitude [47], and is the most commonly used measure [10]. Inter-rater reliability for the proprioception test was excellent [ICC 3, 1: (0.92), (*p* < 0.001)] [48].

### Secondary outcomes

#### Balance

The Romberg test was used to assess balance. Participants were asked to stand erect and were observed by the examiner for 1 minute to reduce ceiling effects. The participants were tested in four conditions: two each on a stable and an unstable surface. The conditions were single-leg standing with eyes open (SEOS, SEOUS) and with eyes closed (SECS, SECUS) [49]. The starting position for participants was standing on the dominant leg, as determined by the football kicking test [50], while the other leg was positioned with the hip in neutral, 90° knee flexion, and arms crossed at chest level. For each condition, the individual was asked to remain in this position and the standing time was recorded. This was repeated three times per condition, with a 30-s rest interval between each trial and a one-minute resting interval between either condition (eyes open or eyes closed).

The assessor monitored the participants carefully during the assessment. Postural retention was considered impossible when the arms moved, the stance foot moved, the lifted foot touched the floor, there was large body sway, eyes were open during eyes-closed trials, or there was a loss of balance that required the assessor to physically prevent a fall [51, 52]. Inter-rater reliability for the balance tests was excellent [ICC 3, 1: (0.92–1.00), (*p* < 0.001)] [48].

### Rehabilitative ultrasound imaging

RUSI was used to evaluate the muscle thickness of the TrA and LM muscles. Images of the TrA and LM muscles were acquired with a Color Doppler Ultrasound machine (Ultrasonic/Canada50nl*OP) and a 5-MHz curvilinear/convex array transducer. Measurement was performed at the thickest part of the muscles and measured in the sagittal plane at the same location for each measurement time point. Image acquisition was performed three times in each measurement condition and the mean value was used for analysis.

**Fig. 2:**
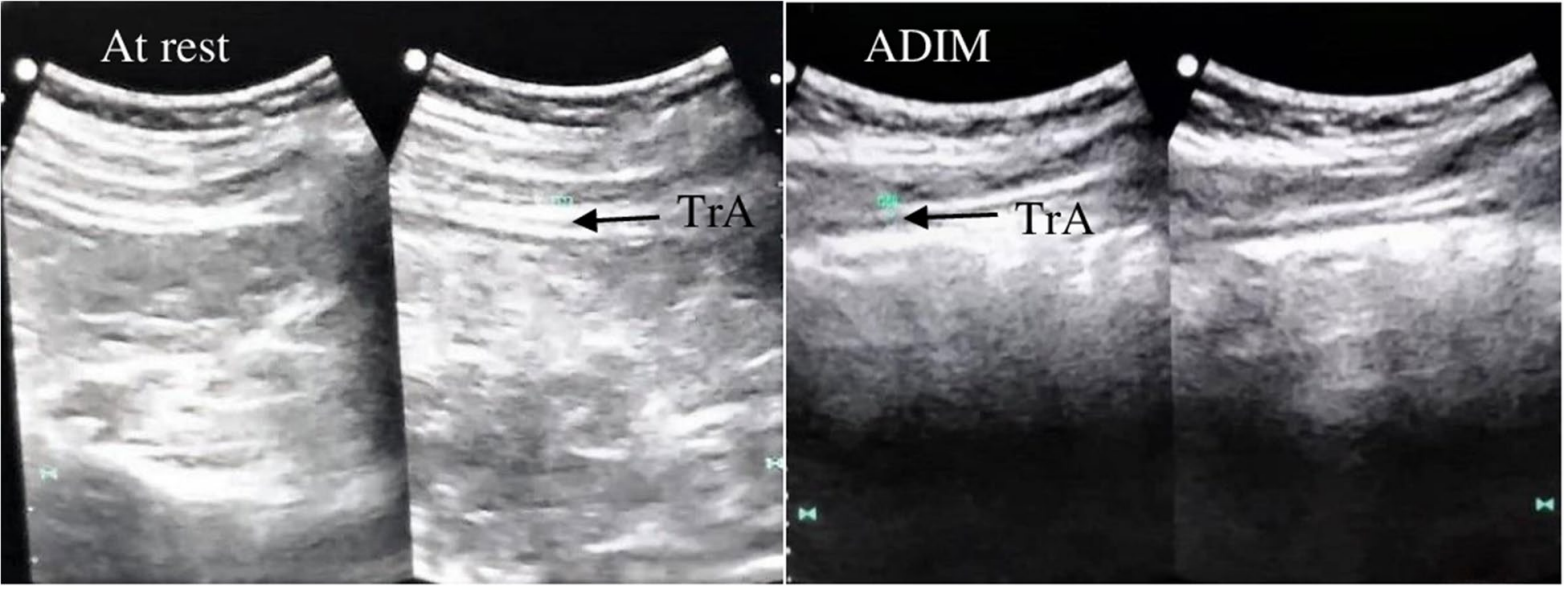
RUSI measurement of the transversus abdominis (TrA) at rest and during abdominal draw in maneuver (ADIM)

The muscle thickness of TrA was measured in two conditions: at rest and during the ADIM (Fig. 2). The participants remained supine in a crook lying position. The transducer was placed along the midaxillary line just superior to the iliac crest, using the measurement protocol described by Teyhen et al. (2005) [53]. TrA thickness was measured between the superficial and deep borders of the muscle, made visible by the hyperechoic fascial lines. To control for the effect of respiration, all images were taken at the end of normal exhalation.

**Fig. 3:**
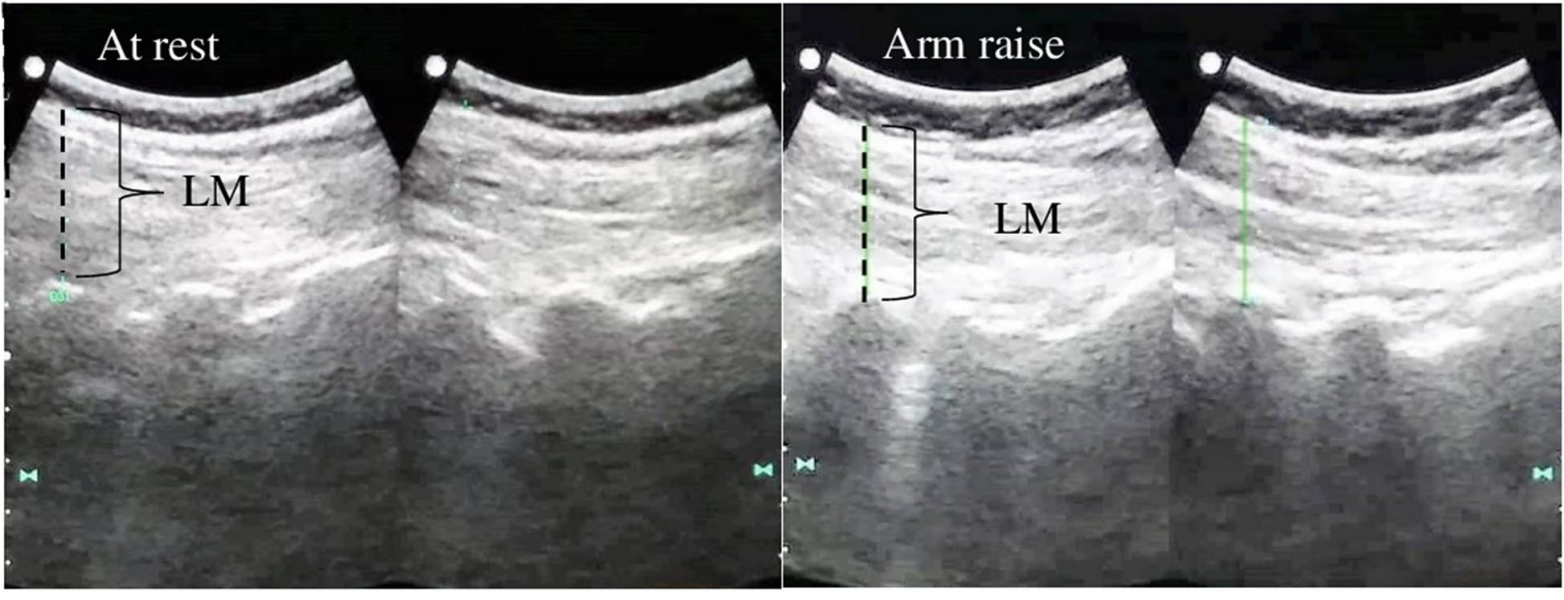
Measurement of the lumbar multifidus (LM) muscle at rest and during a contralateral arm raise

The muscle thickness of the LM muscle was measured in two conditions: at rest and during a submaximal contraction, according to the technique described by Kiesel et al. (2007) [54]. The participants remained in a prone lying position and pillows were placed under their hips to reduce lumbar lordosis. The spinous processes of L_4/5_ were palpated and marked with a pen prior to imaging. The subjects were instructed to relax the paraspinal musculature, electro-conductive gel was applied, and the transducer was placed transversely over the spinous process of L_4/5_. Measurement was taken between the most posterior portion of the L_4/5_ zygapophyseal joint and the inner border of the LM (Fig. 3).

The test-retest reliability of the RUSI measurement for muscle thickness of both sides of the TrA and LM muscles was tested by a senior consultant radiologist with 20 years of work experience (EEK) before the study in patients with subacute NSLBP. The results showed excellent reliability of muscle thickness measurements of TrA [ICC 3, 1: (0.96–0.99), (*p* < 0.001)], and LM [ICC 3, 1: (0.95–0.99), (p < 0.001)], respectively. Muscle thickness at rest, and the percentage of change [(contraction – rest)/ rest ˟ 100] of TrA and LM muscles were determined and analyzed.

### Pain-related outcomes

The visual analogue scale (VAS) was used to assess pain [55], the MODQ was used to measure functional disability [56], and the Tampa Scale for Kinesiophobia (TSK) was used to evaluate fear of movement [57].

### Statistical analysis of the study

For analyzing all the data in this study, STATA version 10.1 (Stata Corp, 4905 Lakeway Drive College Station, Texas 77,845, USA) was used. Demographic data are presented as means (standard deviation) and numbers (percentage). Descriptive statistics, independent sample t-test, and Chi-square tests were used to analyze participant characteristics. Normality of distribution for all data was tested by the Shapiro-Wilk test. The results of this study are presented as mean, standard deviation (SD), and 95% confidence interval (CI). Within-group comparisons on all data were analyzed using the paired t-test. Between-group comparisons were calculated using analysis of covariance (ANCOVA) for adjusting the baseline data. The effect size of the magnitude of standardized difference between groups was calculated using Cohen’s “d” = M_1_-M_2_/ SD_pooled_. *p* < 0.05 was considered a statistically significant level.

## Results

**Fig. 4:**
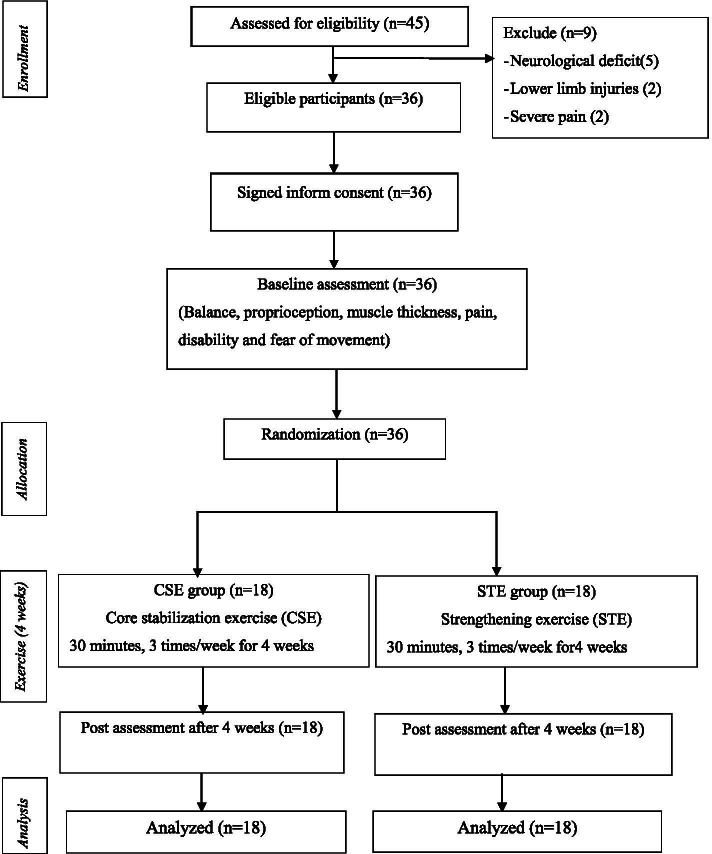
Flow diagram of the participants throughout the study

The demographic and clinical characteristics of participants in both the CSE and STE groups are presented in Table 1. All participants completed the study (Fig. 4). There was no significant difference between groups in relation to demographic and clinical characteristics except fear of movement.

**Table 1:** Baseline demographic and clinical characteristics of the participants

Characteristics	CSE (***n*** = 18)	STE (***n*** = 18)	***p***-value
**Age (years)**	35.06 ± 9.55	34.50 ± 8.83	0.857
**Sex**			0.171
Male	5 (27.78%)	9 (50%)	
Female	13 (72.22%)	9 (50%)	
**Weight (kg)**	60.79 ± 12.78	63.24 ± 9.87	0.165
**Height (cm)**	158.58 ± 8.22	162.08 ± 6.49	0.525
**Body mass index (kg/m**^**2**^**)**	24.02 ± 3.15	24.04 ± 3.34	0.878
**Side of pain**			0.360
Left	4 (22.22%)	8 (44.44%)	
Right	5 (27.78%)	4 (22.22%)	
Both	9 (50%)	6 (33.33%)	
**Duration of current pain (weeks)**	8.33 ± 1.57	8.11 ± 1.68	0.684
**Proprioception (JRE) (cm)**	0.62 ± 0.22	0.69 ± 0.30	0.399
**Balance**			
**SEOS (sec)**	27.05 ± 13.75	26.52 ± 12.92	0.906
**SECS (sec)**	3.13 ± 1.66	3.81 ± 2.92	0.397
**SEOUS (sec)**	9.35 ± 10.05	9.18 ± 8.11	0.956
**SECUS (sec)**	1.56 ± 0.55	1.34 ± 0.25	0.154
**Muscle thickness (RUSI)**			
**Left TrA - at rest (cm)**	0.24 ± 0.05	0.24 ± 0.07	0.935
**- % change**	50.82 ± 16.81	54.29 ± 22.98	0.609
**Right TrA - at rest (cm)**	0.23 ± 0.06	0.23 ± 0.05	0.797
**- % change**	50.47 ± 21.57	56.91 ± 24.94	0.413
**Left LM - at rest (cm)**	2.56 ± 0.50	2.83 ± 0.61	0.146
**- % change**	18.44 ± 10.71	14.27 ± 6.85	0.174
**Right LM - at rest (cm)**	2.52 ± 0.48	2.76 ± 0.50	0.295
**- % change**	17.60 ± 7.99	19.73 ± 8.61	0.447
**Pain (VAS) (cm)**	4.28 ± 1.13	4.61 ± 1.20	0.395
**Functional disability (MODQ) (score /100)**	39.89 ± 10.48	42.89 ± 10.25	0.405
**Fear of movement (TSK) (score /68)**	42 ± 4.16	44.89 ± 3.94	0.040

Values are presented as the mean ± SD and numbers (%). *SEOS, SECS, SEOUS, SECUS* Single leg standing eyes open and eyes closed on stable and unstable surface, *JRE* Joint repositioning error, *VAS* Visual analog scale, *MODQ* Modified Oswestry disability questionnaire, *TSK* Tampa Scale for Kinesiophobia, *RUSI* Rehabilitative Ultrasound Imaging, *TrA* Transversus abdominis, *LM* Lumbar multifidus

The within group analysis demonstrated a significant reduction of the primary outcome, joint repositioning error, in both exercise groups after 4 weeks of intervention (*p* < 0.001). Significant improvements were demonstrated in balance: single-leg standing with eyes open and eyes closed on both stable and unstable surfaces (*p* < 0.001), percentage change of muscle thickness of TrA and LM (*p* < 0.001), and reduction of pain, functional disability and fear of movement in both the CSE and STE groups from baseline to 4 weeks of intervention was observed (*p* < 0.001).

Comparisons between the two exercise groups are shown in Table 2. After 4 weeks of intervention, the CSE group demonstrated significantly reduced joint reposition error compared with the STE group (*p* < 0.001). The CSE group was superior to the STE group in improving balance: single-leg standing with eyes open and eyes closed, on both stable and unstable surfaces, after 4 weeks of intervention (*p* < 0.05). Additionally, significant improvement was found in the percentage change of muscle thickness on both sides of TrA (*p* = 0.001) and LM (*p* < 0.01) in the CSE group compared with the STE group. Conversely, there was no significant difference revealed in the muscle thickness of TrA and LM at rest (*p* > 0.05).

**Table 2:** Comparison between core stabilization exercise and strengthening exercise after intervention

Variables	Adjusted Mean		Adjusted differences between groups, (95% CI)^b^	***P***-value	Effect size
	CSE group (n = 18)	STE group (n = 18)			
**JRE (cm)** ^**a**^	0.17	0.47	−0.30 (− 0.37 to − 0.2)	< 0.001*	1.38
**Balance**					
**SEOS (sec)**	40.85	34.44	6.41 (1.12 to 11.72)	0.019*	0.83
**SECS (sec)**	7.69	5.39	2.30 (0.24 to 4.36)	0.030*	0.67
**SEOUS (sec)**	23.92	15.62	8.30 (0.81 to 15.79)	0.031*	0.76
**SECUS (sec)**	2.47	1.97	0.50 (0.08 to 0.92)	0.022*	0.82
**Muscle thickness (RUSI)**					
**Left TrA - at rest (cm)**	0.27	0.26	0.01 (−0.01 to 0.02)	0.571	0.15
**- % change**	73.24	57.57	15.66 (7.27 to 24.05)	0.001*	1.29
**Right TrA - at rest (cm)**	0.26	0.25	0.01 (−0.01 to 0.02)	0.401	0.39
**- % change**	74.49	60.62	13.86 (6.59 to 16.14)	< 0.001*	1.30
**Left LM - at rest (cm)**	2.81	2.80	0.01 (−0.06 to 0.08)	0.771	0.11
**- % change**	21.50	18.10	3.41 (1.59 to 5.22)	0.001*	1.39
**Right LM - at rest (cm)**	2.73	2.67	0.05 (−0.01 to 0.12)	0.116	0.53
**- % change**	24.97	20.60	4.37 (1.81 to 6.93)	0.001*	1.21
**VAS (cm)** ^**a**^	1.16	1.84	−0.68 (−1.49 to 0.12)	0.093	0.28
**MODQ (score /100)** ^**a**^	13.55	20.56	−7.01 (−12.25 to − 1.77)	0.010*	0.61
**TSK (score /68)** ^**a**^	37.93	40.96	−3.02 (−4.71 to −1.33)	0.001*	0.80

^a^Negative score means improvement in JRE, VAS, MODQ and TSK

*SEOS, SECS, SEOUS, SECUS* Single leg standing eyes open and eyes closed on stable and unstable surface, *JRE* Joint repositioning error, *VAS* Visual analog scale, *MODQ* Modified Oswestry disability questionnaire, *TSK* Tampa Scale for Kinesiophobia, *RUSI* Rehabilitative Ultrasound Imaging, *CI* confidence interval, *TrA* Transversus abdominis, *LM* Lumbar multifidus

^b^Mean differences between groups (95% CI) analyzed by Analysis of Co-Variance (ANCOVA)

Statistically significant difference between 2 groups was set at *p* < 0.05*

When comparing the two exercise groups, the CSE group demonstrated significantly reduced functional disability (*p* = 0.010) and fear of movement scores (p = 0.001). However, there was no significant difference in pain reduction (*p* = 0.093).

## Discussion

The objective of the present study was to investigate the effects of CSE and STE on proprioception, balance, muscle thickness of TrA and LM, and pain-related outcomes in patients with subacute NSLBP after partaking in 4 weeks of hospital-based, individualized training. The results of the study revealed that the proprioception outcome of the CSE group was superior to that of the STE group in patients with subacute NSLBP.

Local trunk muscle spindle dysfunction may decrease low back proprioception resulting in motion error [58] and motor function deficit [18]. After 4 weeks of training, the CSE group demonstrated greater improvement in proprioception than the STE group. During CSE, emphasis was placed on retraining TrA and LM muscles, which possibly increased the muscle activity of those muscles, and stimulating muscle spindles and joint receptors, thereby improving the accuracy of the sensory motor integration procedure and initiating precise joint repositioning [38, 39]. The results of the current study align with those of Puntumetakul et al. (2018), the sole study that has explored the effect of CSE on proprioception in patients with subacute NSLBP. It demonstrated that a CSE program could reduce pain and improve proprioception [36].

The between-group analysis demonstrated that the CSE group was superior to the STE group in terms of balance control after 4 weeks of intervention. Re-educating of TrA and LM muscles, which have rich sources of sensory input and play an important role in providing postural control and enhancing balance control [30]. Therefore, CSE may help patients to recover from their neuromuscular dysfunction, improve the somatosensory processes that restore kinesthetic awareness, improve proprioception, and enable relearning of finely-tuned spinal control [38, 39, 59, 60].

The results of the present study are similar to those reported by Choi and coworkers (2018). These researchers ascertained the effects of a core exercise program on balance in patients with chronic LBP and proposed that it improved balance abilities in patients with chronic LBP [61]. The results of the present study differed from the previous findings of Shamsi et al. (2017), who reported no significant difference in stability indices between the two exercise groups in patients with chronic NSLBP [41]. Additionally, Puntumetakul and coworkers (2020) determined that both CSE and STE exercises could improve balance performance and reduce pain intensity in chronic LBP patients with clinical lumbar instability [62]. These contrasting findings may be due to the heterogeneity of exercise frequency and duration, method of assessment, and stage and condition of LBP.

The present findings demonstrated that the CSE group had a more significant improvement in the percentage change of muscle thickness on both sides of the TrA and LM when compared with the STE group. This may be due to CSE being based on the motor learning approach, with a focus on the preferential recruitment of TrA and LM muscles, which could reverse pain-related restructuring in the motor cortex, and reinforce the integration of motor control and enhanced muscle behavior [26].

CSE can increase muscle thickness, the activity of LM muscles, and motor control capacity in patients with LBP [26, 63, 64]. Previous investigators have postulated that CSE with ADIM can minimize compensation, effectively increase abdominal muscle thickness and improve balance and spinal stability [42, 65–67]. The results of the present study support these findings: the CSE program may increase the percentage change of muscle thickness of the TrA and LM muscles.

On the other hand, there was no significant difference found in the muscle thickness of TrA and LM at rest between the CSE and STE groups after 4 weeks of intervention. The exercise duration for the present study was only 4 weeks, which may have been an insufficient duration to show significantly different effects on muscle thickness at rest between the two exercise groups. A recent randomized clinical trial compared the McKenzie method of exercises with motor control exercises and reported that the two types of exercises similarly improved abdominal muscle thickness [68], which aligns with the present study’s results.

There was no significant difference in reduction of pain between the CSE and STE groups after 4 weeks of intervention. However, the number of subacute NSLBP patients who totally recovered from pain was 38.89% in the CSE group and 16.67% in the STE group. Functional disability was more significantly reduced in the CSE group after 4 weeks of intervention when compared with the STE group. This may be because CSE improves activation and coordination of the trunk muscles, enhances the stability of the lumbar segment, and reduces spinal overload, pain, and functional disability. Similar to previous studies results (Kong et al. (2015) and Byström et al. (2013)), we propose that CSE can improve back movement performance by reducing pain, which may improve the accuracy of joint repositioning sense and reduce functional disability [39, 69].

After 4 weeks of intervention, both the CSE and STE groups had significantly reduced fear of movement. The CSE program was superior to the STE program in terms of fear reduction. This may be because CSE aims to maintain neutral spinal posture, activates the deep muscles with minimal activity of the superficial muscles, uses low-load activities that improve muscle coordination, and decreases muscle spasm and tension. One previous observational study revealed that trunk stiffness was positively correlated with fear of movement in patients with LBP [70]. We postulate that CSE can provide fine-tuning control that eliminates the trunk stiffening strategy, decreases the compressive load on the spine, and thus reduces pain, functional disability and fear of movement in patients with subacute NSLBP.

The limitations of the present study are as follows. This study examined only in patients with subacute NSLBP, and further trials should explore the effect of CSE in subacute NSLBP patients with and without lumbar instability. Participants’ criteria of this study were limited with regards to age “50 years old; mean age, 34.78±9.07 years” and with regards to pain “moderate level of pain (3/10 - 7/10); mean pain level, 4.44±1.16 scores”. Further studies should be conducted in older age groups and higher level of pain. Although the sample size in this study was small, the effect size between CSE and STE groups for our primary outcome (proprioception) was 1.38, which is very large [71, 72]. The choice of using the ADIM to measure the TrA activation outcome may favor participants in the CSE group as the ADIM, which is under volitional control, was part of the CSE training, in contrast to the STE group. Additionally, the individuals responsible for training the CSE and STE groups were fundamentally different (one researcher vs experienced physical therapists, respectively) which, may have resulted in bias. Exercise compliance was not tested and as such may be different between the CSE and STE groups, introducing a bias. Moreover, RUSI in the present study only assessed the muscle thickness of deep trunk muscles. Future trials should assess the muscle thickness of both deep and superficial muscles. There was more fear of movement in the STE group before intervention, may have a confounding effect. Therefore, this result needs to be interpreted with caution.

## Conclusion

This study supports CSE as an optimal treatment for improving proprioception, balance, and percentage change of muscle thickness while reducing functional disability and fear of movement in patients with subacute NSLBP.

## Supplementary Information

**Additional file 1: Appendix 1.** Core stabilization exercise Program. **Appendix 2–** Strengthening exercise Program. The procedure for core stabilization exercises and strengthening exercise comprises of diagrams of each exercise for the 4 week exercise program. Intensity of exercise is based on the participant exercise performance. All exercises involve a hold for 10 s with 10 repetitions. Each exercise session is performed for 30 min, with three sessions per week.

## Data Availability

The authors will allow sharing of participant data, such as proprioception, balance and muscle thickness. The data will be available for anyone who wishes to access them for any purpose. The data will be accessible from immediately following publication to 6 months after publication, and contact should be made via the Corresponding author rungthiprt@gmail.com.

## Abbreviations

ADIM: Abdominal drawing-in maneuver
ANCOVA: Analysis of Co-Variance
CI: Confidence interval
CSE: Core stabilization exercise
d: Effect size
ICC: Intraclass correlation coefficient
JRE: Joint repositioning error
LBP: Low back pain
LM: Lumbar multifidus
M_1_: Mean_group 1_
M_2_: Mean_group 2_
MODQ: Modified Oswestry Disability Questionnaires
NSLBP: Nonspecific low back pain
n: Sample size
RUSI: Rehabilitative ultrasound imaging
SD: Standard deviation
SD_pooled_: Pooled standard deviation
SEOS, SECS, SEOUS, SECUS: Single leg standing eyes open and eyes closed on stable and unstable surface
STE: Strengthening exercise
TSK: Tampa Scale for Kinesiophobia
TrA: Transversus abdominis
VAS: Visual Analog Scale
