# Supplementary material for: Effects of core stabilization exercise and strengthening exercise on proprioception, balance, muscle thickness and pain related outcomes in patients with subacute nonspecific low back pain: a randomized controlled trial
Source: BMC Musculoskelet Disord. 2021 Nov 30;22:998. doi: 10.1186/s12891-021-04858-6 (PMC8630919; doi:10.1186/s12891-021-04858-6)
Supplement: Supplementary file 1 — Additional file 1: Appendix 1. Core stabilization exercise Program. Appendix 2– Strengthening exercise Program. The procedure for core stabilization exercises and strengthening exercise comprises of diagrams of each exercise for the 4 week exercise program. Intensity of exercise is based on the participant exercise performance. All exercises involve a hold for 10 s with 10 repetitions. Each exercise session is performed for 30 min, with three sessions per week. [file 12891_2021_4858_MOESM1_ESM.docx]

**Additional file 1**

**Appendix 1– Core stabilization exercise Program**

Instruction: This document shows the procedure for core stabilization exercises, it comprises of diagrams of each exercise for the four weeks exercise program. Arrows indicate the required specific trunk muscle activation in this exercise. Intensity of exercise is based on the participant exercise performance. All exercises involve a hold for 10 seconds with 10 repetitions. Each exercise session is performed for 30 minutes, with three sessions per week, and daily home exercises should be performed following these instructions.

| **Week** | **Exercise procedure of Core stabilization exercise** |
| --- | --- |
| **Week one- Isolated transversus abdominis and lumbar multifidus training** | |
| 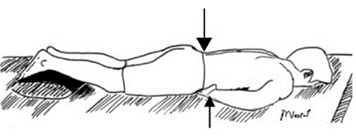  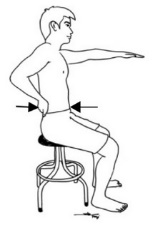 | **Transversus abdominis**  In prone lying position on a couch with a small pillow placed beneath their ankles. Participants are asked to gently draw in the lower anterior abdominal wall (ADIM) with normal breathing control, no movement of the spine and pelvis. |
|  |  |
|  |  |
|  |  |
|  | **Lumbar multifidus**  Sit on the chair and LM activation will be stimulated by raising the contralateral arm while performing ADIM. |
|  |  |
|  |  |
| **Week two- Co-contraction of transversus abdominis and lumbar multifidus** | |
|    | **In the sitting position**  1. Perform co-contraction of TrA and LM (ADIM) in the sitting position on a chair. Use the index and middle fingers to palpate contraction of transversus abdominis muscle and the two fingers of the opposite hand to palpate contraction of lumbar multifidus muscle.  2. Train forward and backward trunk movements keeping the lumbar spine and pelvis in a neutral position. |
|  |  |
|  |  |
|  |  |
|  | **In the lying position**  Perform co-contraction of the two muscles in a crook lying position with both hips at 45 degrees and both knees at 90 degrees.   1. Abduct one leg to 45 degrees. 2. Slide a single leg down until the knee is straight. |
|  |  |
|  |  |
| **Week three- Co-contraction of transversus abdominis and lumbar multifidus** | |
| 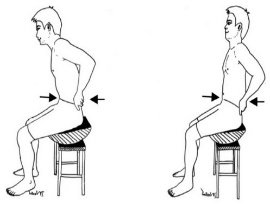 | **In the sitting position**  Train co-contraction of the two muscles while sitting on a balance board.  1. Forward, backward and sideways movements of the trunk while keeping spine in a neutral position. |
|  |  |
|  |  |
|  |  |
|  | **In the lying position**  Performing co-contraction of the two muscles in the crook lying position.   1. Raise the buttocks off the bed. 2. Repeat above exercise with one leg crossed over the supporting leg. |
|  |  |
|  |  |
| **Week four- Co-contraction of transversus abdominis and lumbar multifidus** | |
|     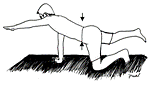   | **In Quadruped position**  Co-contraction of the two muscles is performed in a four-point kneeling position, keeping the back in a neutral position.   1. Raising a single leg. 2. Raising an arm and opposite leg. |
|  |  |
|  |  |
|  |  |
|  | **In standing position**  Co-contraction of the two muscles in a standing position.   1. Flex the hip and knee of one leg to 90 degrees while co-contracting muscles with a ball behind back. 2. Perform ankle movement in forward-backward direction, with both feet on floor. |
|  |  |
|  |  |

**Appendix 2– Strengthening exercise program**

Instruction: This document shows the procedure of strengthening exercise with diagrams of each exercise for the four weeks exercise program. Exercise intensity is based on the participant exercise performance. All exercises are held for 10 seconds for 10 repetitions. Each exercise session is performed for 30 minutes, three sessions per week, and daily home exercises should follow these instructions.

| **Week** | **Exercise procedure of Strengthening exercise** |
| --- | --- |
| **Week one- Specific abdominal and back exercise in lying position** | |
| 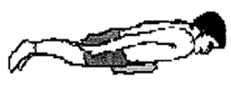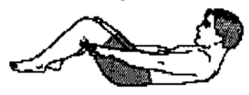 | Train upper abdominals from lying position. Participants are asked to do a partial sit-up from crook lying position (knees bent). |
|  |  |
|  |  |
|  |  |
|  | Train back extensors by lifting their trunk to neutral from prone position with pillow under stomach and arms placed by the sides. |
|  |  |
|  |  |
| **Week two- Abdominal and back exercise with limb movement in lying position** | |
| 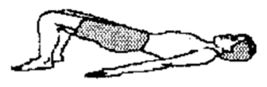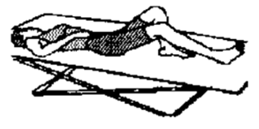 | Train lower abdominals from lying position. The participants will slide their heel slowly. |
|  |  |
|  |  |
|  |  |
|  | Train back extensors by bridging and lifting their trunk to neutral from supine lying position. |
|  |  |
|  |  |
| **Week three- Abdominal exercise in side lying position and back exercise in 4-point kneeling position position** | |
| 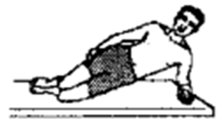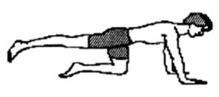 | Obliques abdominis will be trained in hip lift from side-lying position. |
|  |  |
|  |  |
|  |  |
|  |  |
|  | For training back extensors, participants will be asked to perform single-leg extensions from four-point kneeling position. |
|  |  |
|  |  |
| **Week four- Abdominal exercise in supine lying position and back exercise in 4-point kneeling position position** | |
| 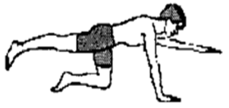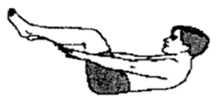 | The participants will be asked to train abdominals from lying position and to perform full abdominal crunches. |
|  |  |
|  |  |
|  |  |
|  | For training back extensors, the participants will be asked to perform alternate arm and leg lifting from four-point kneeling position. |
|  |  |
|  |  |
